# Supplementary material for: The PripA-TbcrA complex-centered Rab GAP cascade facilitates macropinosome maturation in Dictyostelium
Source: Nat Commun. 2022 Apr 4;13:1787. doi: 10.1038/s41467-022-29503-1 (PMC8980073; doi:10.1038/s41467-022-29503-1)
Supplement: Supplementary file 1 — Supplementory Information [file 41467_2022_29503_MOESM1_ESM.pdf]

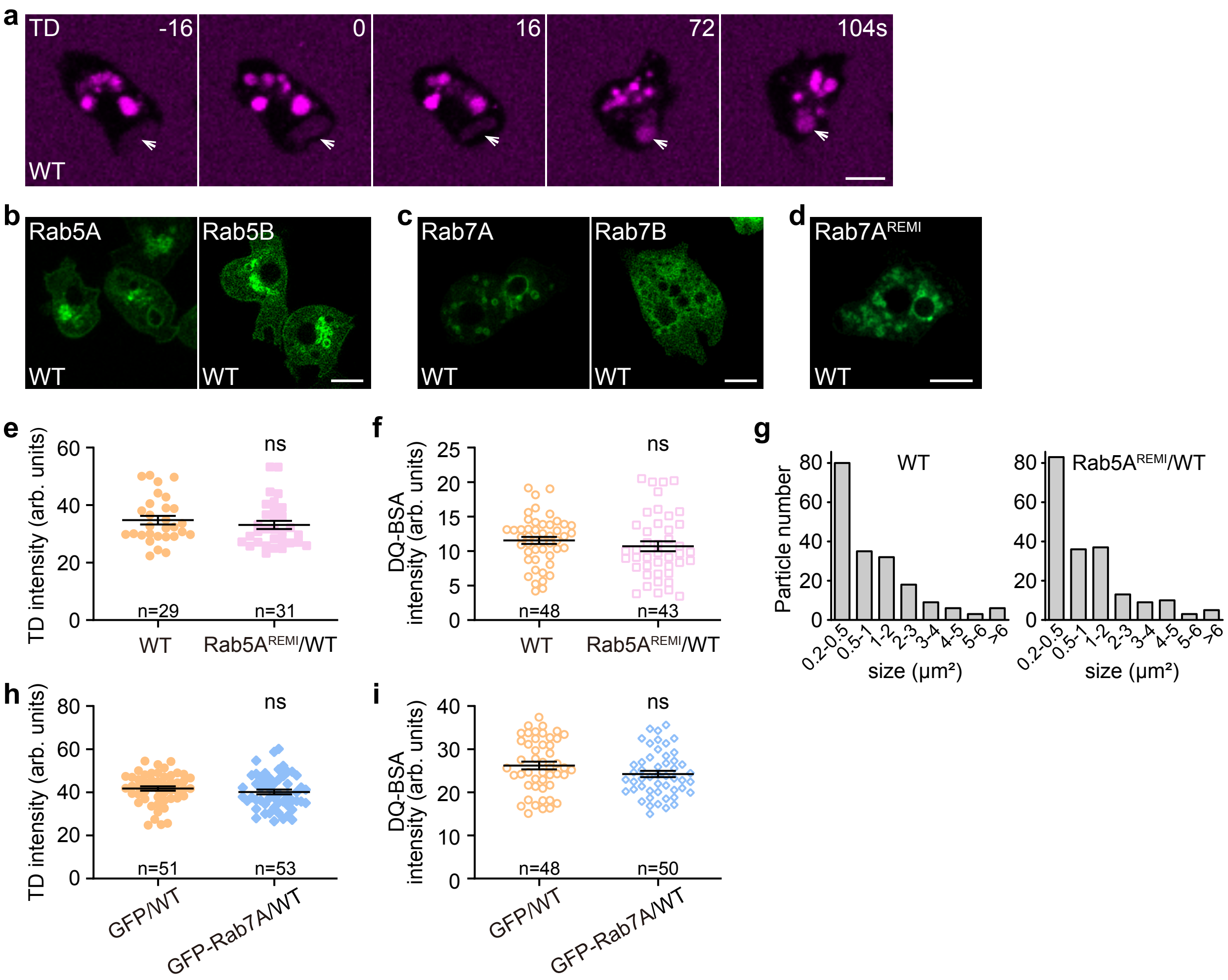

Figure S1. Localization of Rab5 and Rab7. (a) Time-lapse images of TD uptake. The arrows mark a newly formed macropinosome that shrunk over time. (b-c) Localization of GFP-tagged Rab5A, Rab5B, Rab7A, and Rab7B expressed from extrachromosomal vectors in WT cells. (d) Localization of GFP-Rab7A expressed from a stable copy integrated into the WT genome via REMI. (e-f) Quantification of TD uptake and DQ-BSA degradation in WT and GFP-Rab5A^{REMI}/WT cells. (g) Quantification of the size distribution of TD-containing macropinosomes in WT and GFP-Rab5A^{REMI}/WT cells. (h-i) Quantification of TD uptake and DQ-BSA degradation in GFP/WT and GFP-Rab7A/WT cells. Images were acquired after a 30-min incubation with TD or DQ-BSA. The scatter plots show data points with means and SEM. Significance was determined by two-tailed unpaired t test with Welch's correction.Scale bar = 5 μm. Source data for e-i are provided in this paper.

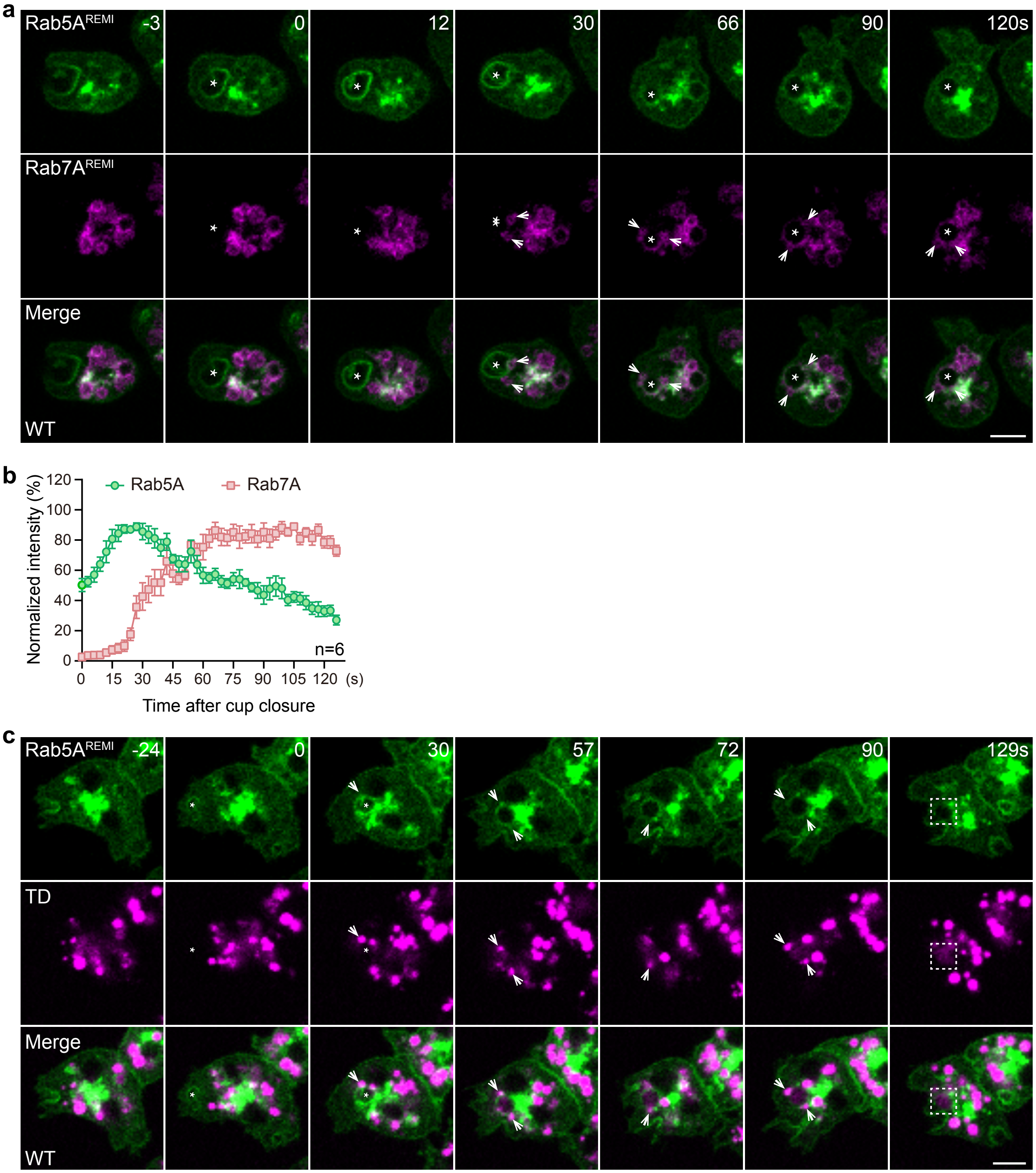

Figure S2. Rab5-to-Rab7 conversion during macropinocytosis. (a) Time-lapse images of macropinocytosis in WT cells expressing GFP-Rab5A and RFP-Rab7A from genome-integrated expression cassettes. The asterisks mark a newly enclosed macropinosome initially decorated by GFP-Rab5A^{REMI}. The arrows mark RFP-Rab7A^{REMI}-labeled late macropinosomes that surrounded and fused with the nascent macropinosome. (b) Quantification of the normalized fluorescent intensity changes of GFP-Rab5A^{REMI} and RFP-Rab7A^{REMI} on macropinosomes after cup closure (mean $\pm$ SEM). (c) Time-lapse images of GFP-Rab5A^{REMI}/WT cells pre-incubated with TD for 60 min. Images were acquired after TD was washed out. The arrows mark preformed macropinosomes containing bright TD signal. The asterisks and dashed boxes mark the newly generated macropinosome which gradually obtained TD signal. Scale bar = 5 μm. Source data for b are provided in this paper.

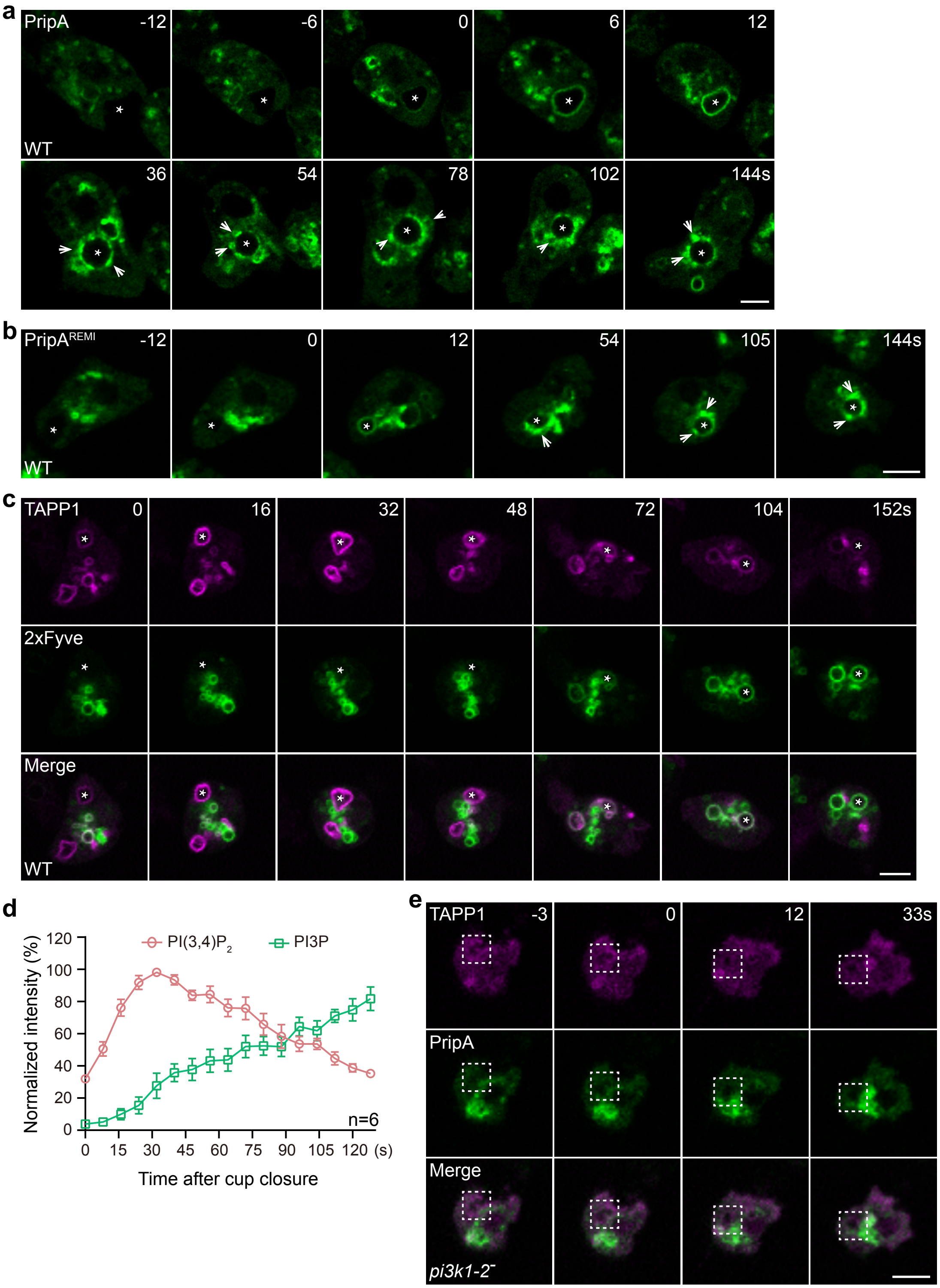

Figure S3. Localization of PripA during macropinocytosis. (a-b) Time lapse images of PripA-GFP expressed in WT cells from extrachromosomal vectors (a) or genome-integrated expression cassettes (b). The asterisks mark newly formed macropinosomes, and the arrows mark vesicles that surrounded and fused with newly formed macropinosomes. (c) Sequential accumulation of TAPP1-RFP and GFP-2×Fyve during macropinocytosis. The asterisks mark newly formed macropinosomes. (d) Quantification of the normalized fluorescent intensity changes of TAPP1-RFP and GFP-2×Fyve on macropinosomes after cup closure (mean ± SEM). (e) Localization of TAPP1-RFP and PripA-GFP in *pi3k1-2⁻* cells during macropinocytosis. The dashed boxes mark newly formed macropinosomes. Scale bar = 5 μm. Source data for d are provided in this paper.

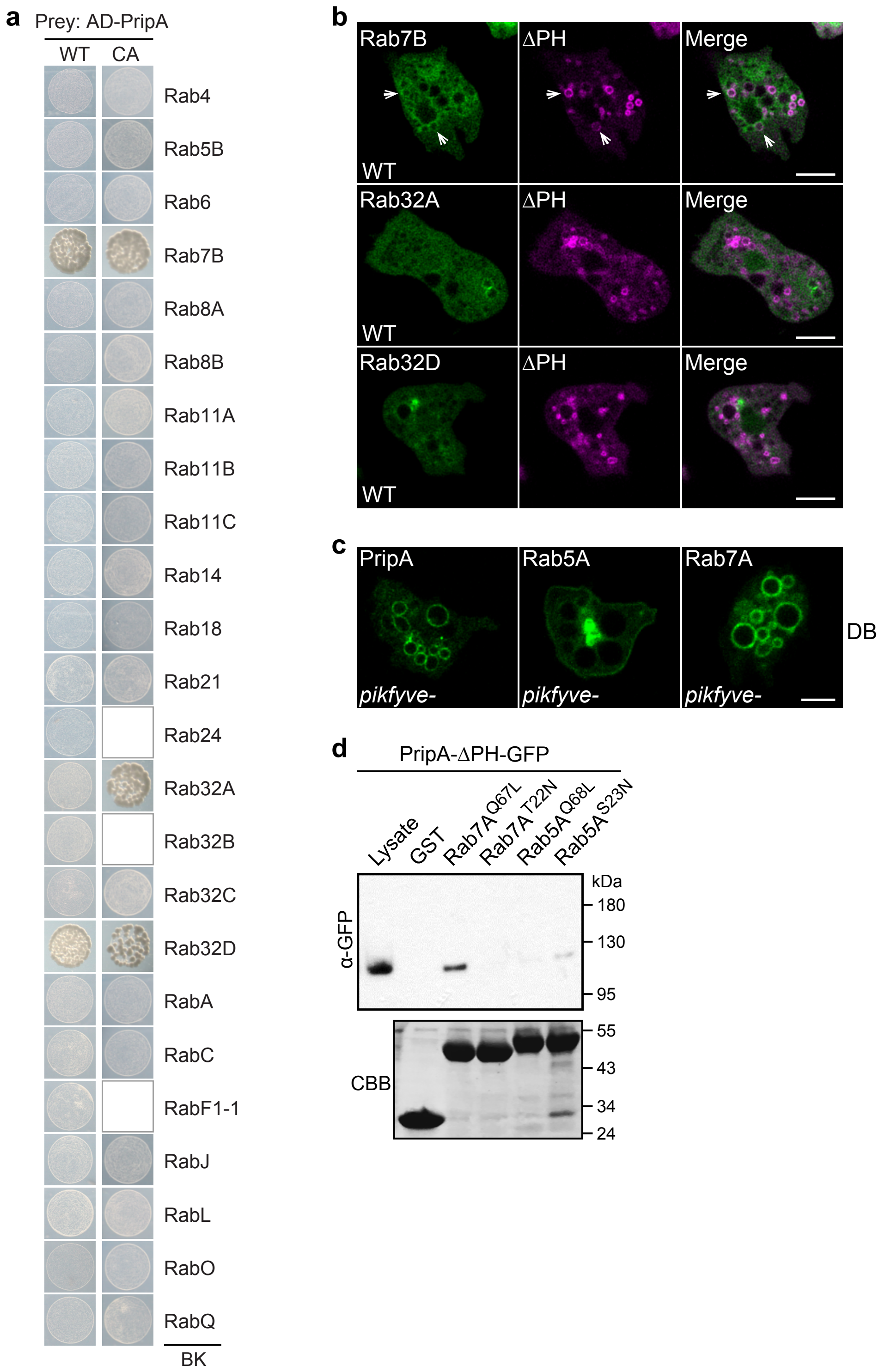

Figure S4. PripA interacts with activated Rab7A. (a) Yeast two-hybrid assay using PripA as prey and 26 Rab GTPases in their respective WT or CA forms as bait. The CA forms of Rab24, Rab32B, and RabF1-1 were not included because these proteins do not contain the conserved glutamine residue that stabilizes Rab proteins in an active conformation. (b) Co-localization of PripA-ΔPH-RFP with GFP-tagged Rab7B, Rab32A, and Rab32D. (c) Localization of PripA-GFP, GFP-Rab5A or GFP-Rab7A in *pikfyve*⁻ cells cultured in developmental buffer (DB) for 30 min. Scale bar = 5 μm. (d) Top: Western blot from pull-down of GST or GST-fused CA or DN forms of Rab5A and Rab7A beads with cell lysate expressing PripA-ΔPH-GFP. Bottom: the protein-transferred membrane was stained with CBB to show purified GST fusion proteins. Data was from one representative experiment out of two independent experiments. Source data for d are provided in this paper.

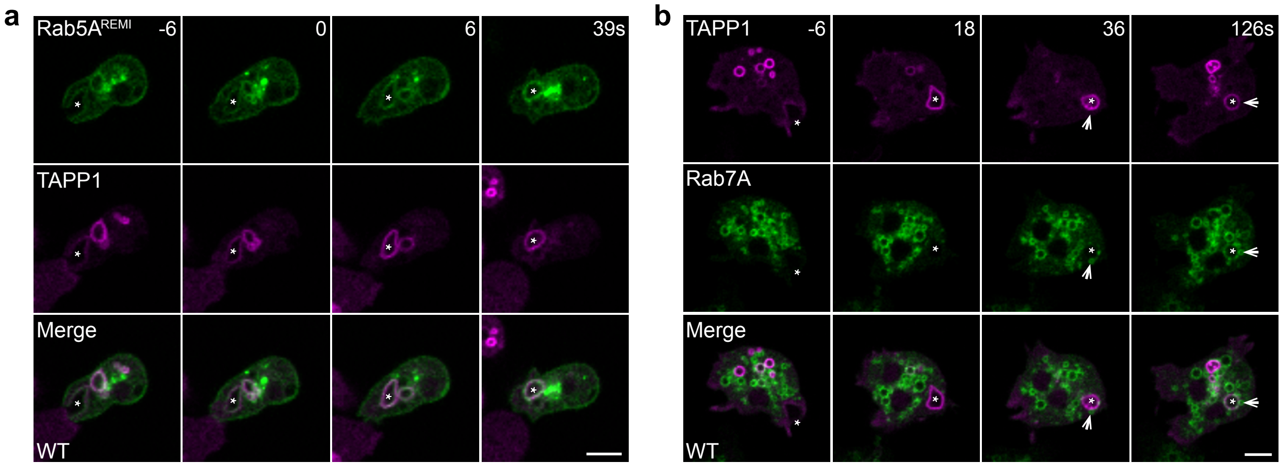

Figure S5. Time-lapse images of protein localization during macropinocytosis. (a) Co-localization of GFP-Rab5A^{REMI} and TAPP1-RFP on nascent macropinosomes. (b) Localization of TAPP1-RFP and GFP-Rab7A. Scale bar = 5 µm.

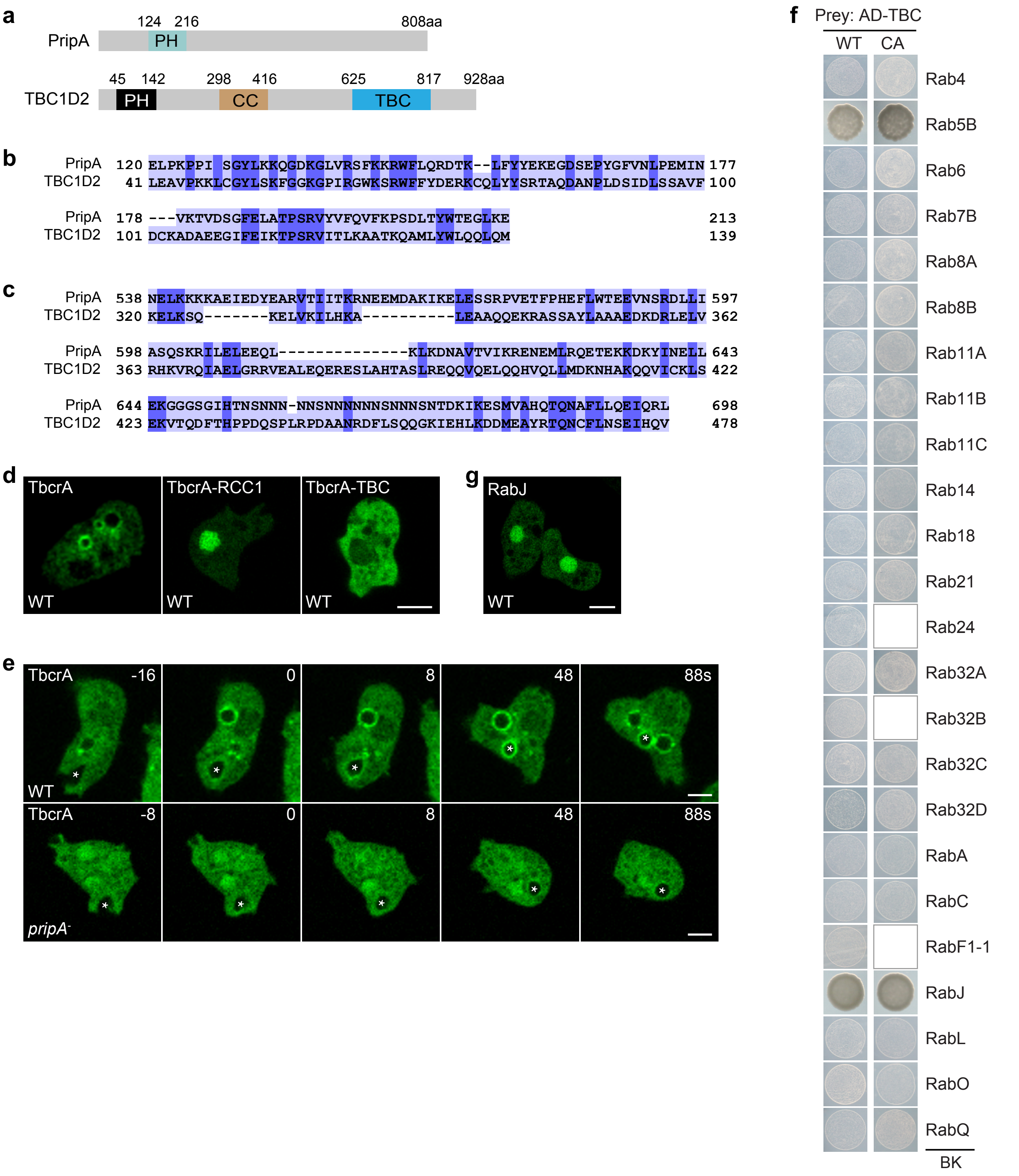

Figure S6. PripA forms a complex with TbcrA, a putative Rab5 GAP. (a) Schematic representation of PripA and human TBC1D2. (b) Sequence alignment of PripA (120-213 aa) and human TBC1D2 (41-139 aa). The sequences span the PH domains in PripA and TBC1D2. (c) Sequence alignment of PripA (538-698 aa) and human TBC1D2 (320-478 aa). (d) Localization of GFP-tagged TbcrA, TbcrA-RCC1, and TbcrA-TBC in WT cells. (e) Localization of GFP-TbcrA in WT and *pripA*$^-$ cells. Nascent macropinosomes are marked by asterisks. (f) Yeast two-hybrid assay using TbcrA-TBC as prey and 26 Rab GTPases in their respective WT or CA forms as bait. The CA forms of Rab24, Rab32B, and RabF1-1 were not included because these proteins do not contain the conserved glutamine residue that stabilizes Rab proteins in an active conformation. (g) Localization of GFP-RabJ in WT cells. Scale bar = 5 μm.

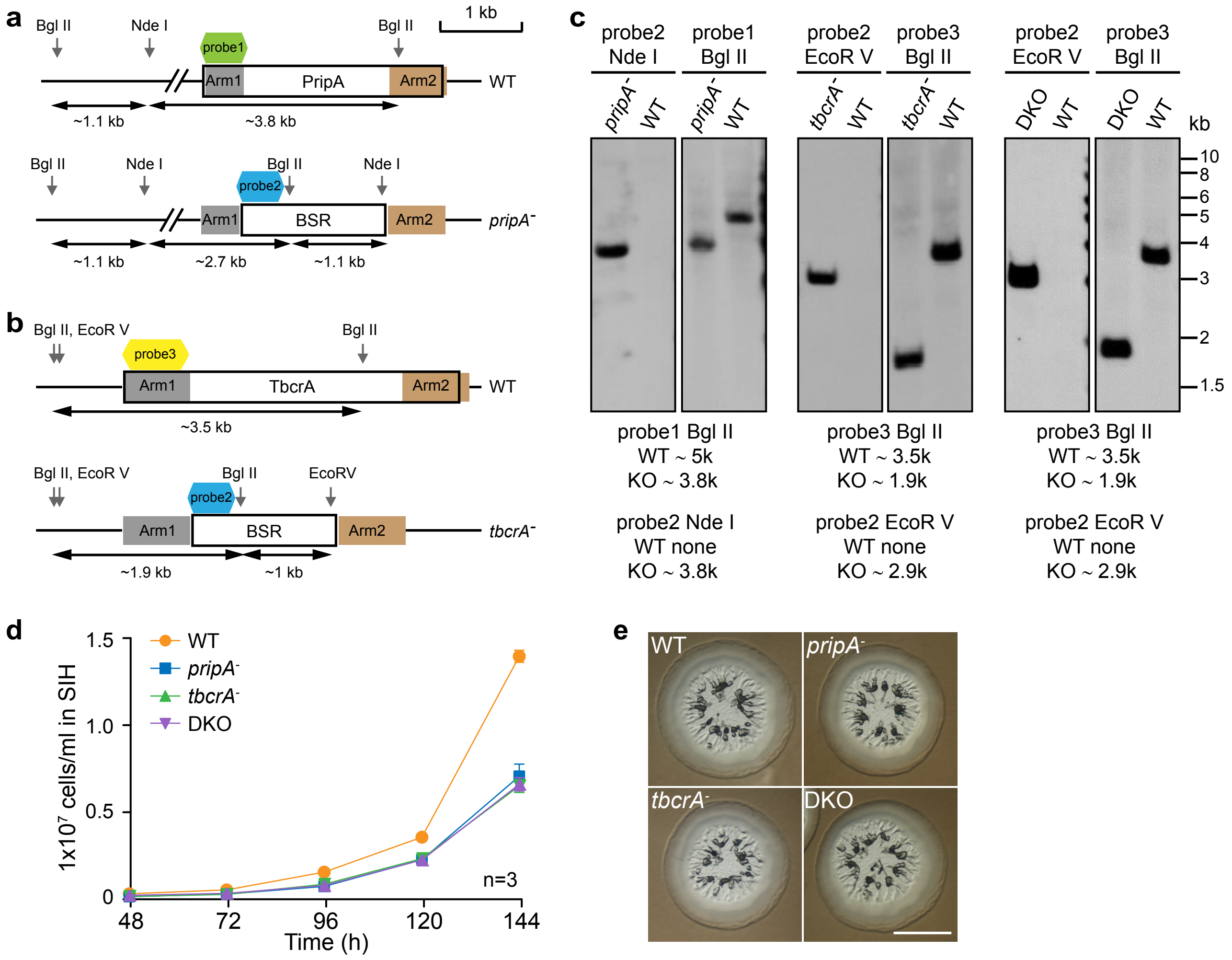

Figure S7. Generation of *pripA*-, *tbcrA*-, and DKO cells. (a-b) Design of the knockout constructs. A blasticidin resistant cassette (BSR) was inserted to replace part of the open reading frame of *pripA* or *tbcrA*. To generate DKO cells, the BSR cassette was first removed from *pripA*- cells, and the *tbcrA* gene was then disrupted. Arrows mark the sites where genomic DNA was digested for Southern blot analysis. (c) Targeted clones were confirmed by Southern blot analysis. (d) Growth curve of the indicated cells cultured in SIH medium in suspension (mean ± SEM). Data was from 3 independent experiments. (e) The indicated cells were plated clonally with bacteria (*Klebsiella aerogenes*) on standard medium agar for 5 days. Scale bar = 5 mm. Source data for c and d are provided in this paper.

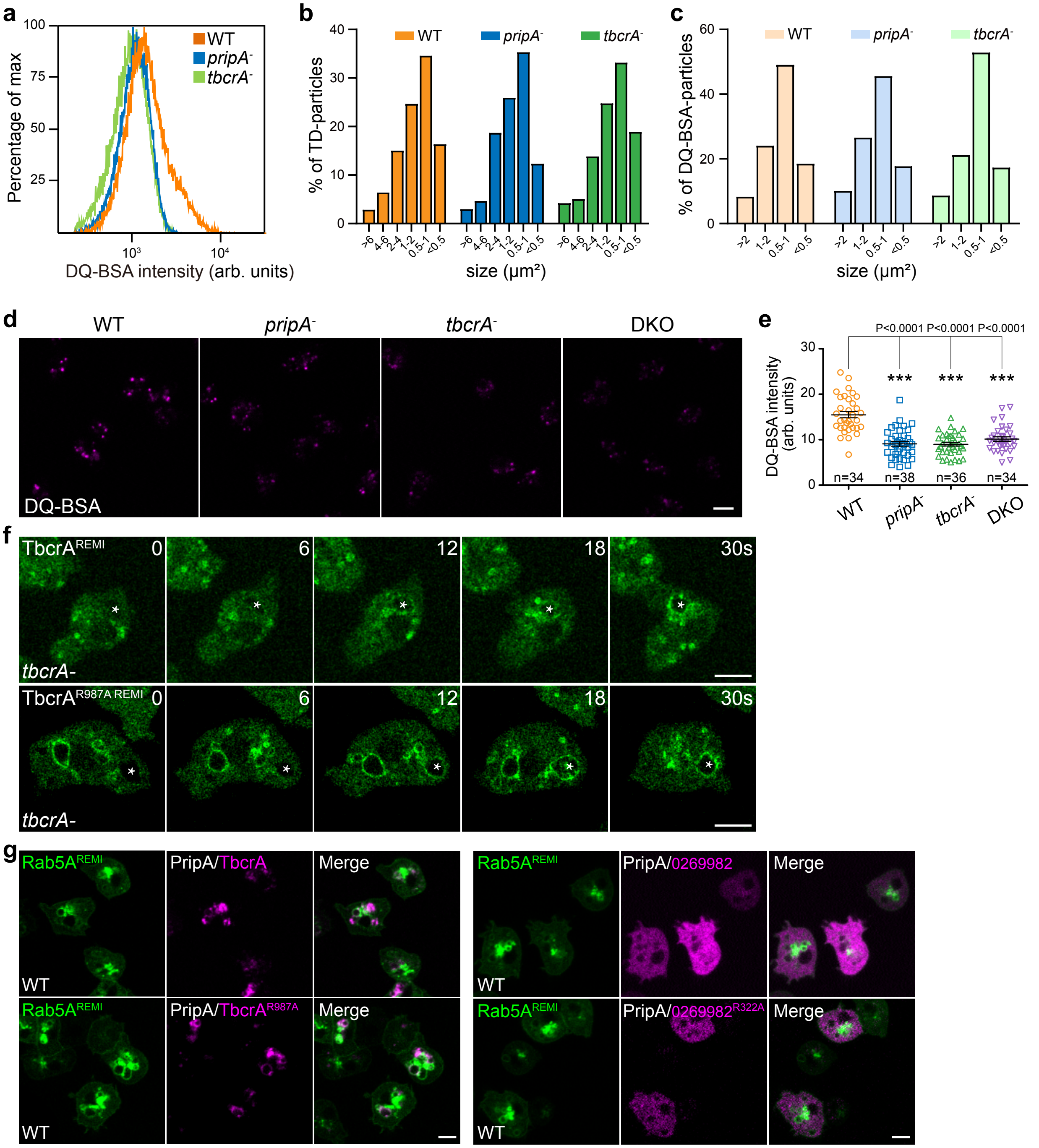

Figure S8. The PripA-TbcrA complex regulates macropinosome maturation. (a) DQ-BSA degradation activity measured by flow cytometry. Cells were incubated with DQ-BSA for 20 min. Each sample represents ~50,000 cells. Data was from one representative experiment out of two independent experiments. (b-c) Quantification of the size distribution of TD- or DQ-BSA-containing macropinocytic vesicles in the indicated cell lines. Images were acquired after a 30-min incubation. (d) Comparison of DQ-BSA degradation in the indicated cell lines. Images were acquired after a 20-min incubation. (e) Quantification of DQ-BSA degradation (means ± SEM). Significance was determined by one-way ANOVA with Dunnett posttest. (f) Localization of GFP-TbcrA and GFP-TbcrA^{R987A} expressed from a stable copy integrated into the genome of *tbcrA*$^-$. (g) GFP-Rab5A^{REMI}/WT cells were transformed with vectors expressing PripA together with RFP-TbcrA, RFP-TbcrA^{R987A}, RFP-DDB_G0269982, or RFP DDB_G0269982^{R322A}. Scale bar = 5 μm. Source data for b, c, and e are provided in this paper.

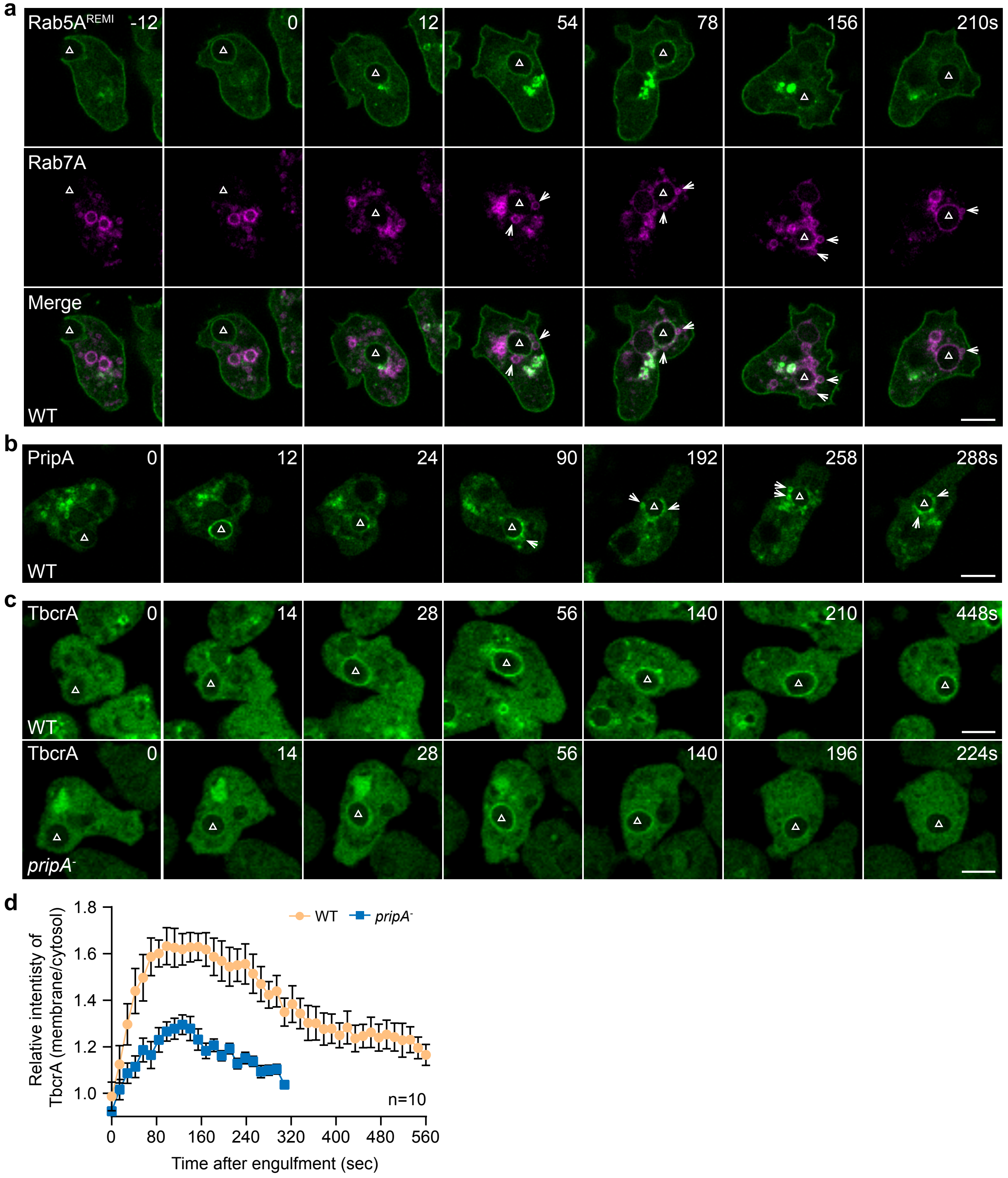

Figure S9. Localization of Rab5, Rab7, PripA, and TbcrA during phagocytosis. (a) Time-lapse images of yeast phagocytosis in GFP-Rab5A^{REMI}/WT cells expressing RFP-Rab7A. The triangles mark a newly formed phagosome initially decorated by GFP-Rab5A^{REMI}. The arrows mark Rab7-labeled vesicles that surrounded and fused with the nascent phagosome. (b) Time lapse images of yeast phagocytosis in WT cells expressing PripA-GFP. The triangles indicate a newly formed phagosome; the arrows mark smaller-sized vesicles labeled by PripA that surrounded the nascent phagosome. (c) Localization of GFP-TbcrA in WT and *pripA*$^{-}$ during phagocytosis. (d) Quantification of the changes of fluorescent intensity of GFP-TbcrA on the phagosomal membrane relative to that in the cytosol (mean ± SEM). Scale bar = 5 μm. Source data for d are provided in this paper.

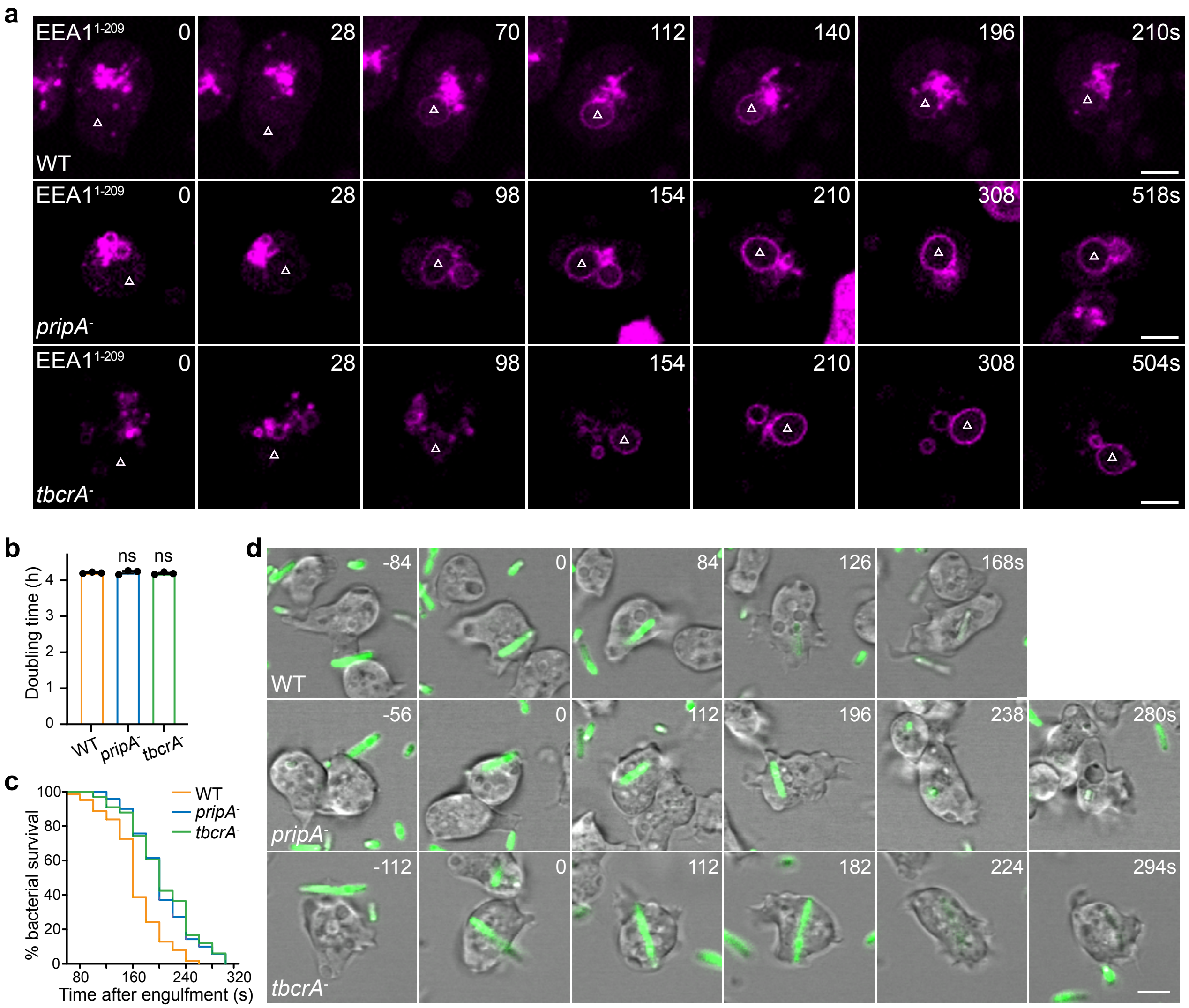

Figure S10. The PripA-TbcrA complex regulates phagosome maturation. (a) Localization of EEA1$^{1\text{-}209}$-RFP in GFP-Rab5A^{REMI}/WT cells or the same cells with deletion of *pripA* or *tbcrA* during phagocytosis. The duration time of EEA1$^{1\text{-}209}$-RFP on phagosomes was 154 ± 61 s in WT, 442 ± 139 s in *pripA*$^-$, and 521 ± 116 s in *tbcrA*$^-$ cells (mean ± SD, n = 6 for WT, n = 15 for *pripA*$^-$, n = 11 for *tbcrA*$^-$). (b) Generation time measured by growing cells in non-nutrient buffer containing live *Klebsiella aerogenes* (mean ± SEM). Data was from 3 independent experiments. Significance was determined by one-way ANOVA with Dunnett posttest. (c) Time-lapse images of cells phagocytosing GFP-expressing *E.coli*. The point of bacterial cell permeabilization and death was inferred from the quenching of GFP fluorescence. (d) Quantification of bacterial survival. The Kaplan-Meyer graph was based on the persistence of bacterial GFP-fluorescence within cells after phagocytosis. At least 60 bacteria were followed in each cell line. Bacteria survived significantly longer in *pripA*$^-$ and *tbcrA*$^-$ cells than WT ($p < 0.001$, Mantel-Cox test). Scale bar = 5 μm. Source data for b and c are provided in this paper.

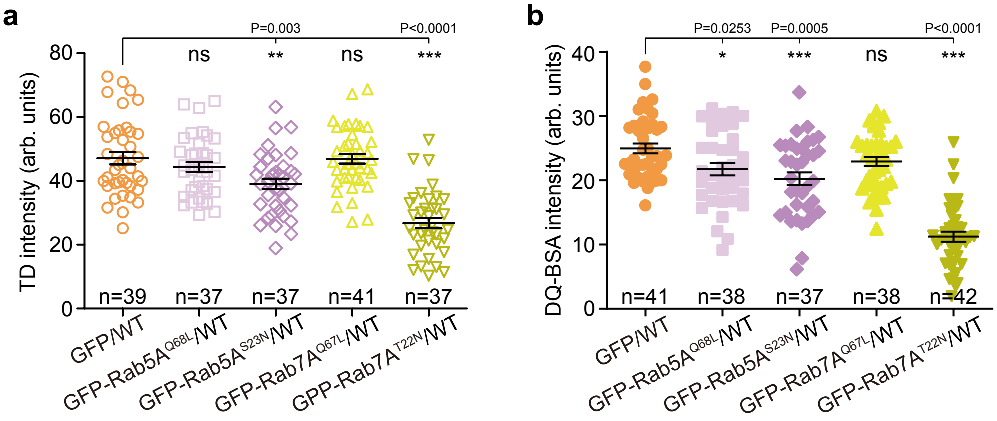

Figure S11. TD uptake and DQ-BSA degradation in WT cells expressing CA or DN form of Rab5A or Rab7A. (a) Quantification of TD uptake. (b) Quantification of DQ-BSA degradation. Images were acquired after a 30-min incubation. The scatter plots show data points with means and SEM. Significance was determined by one-way ANOVA with Dunnett posttest. Source data for a and b are provided in this paper.

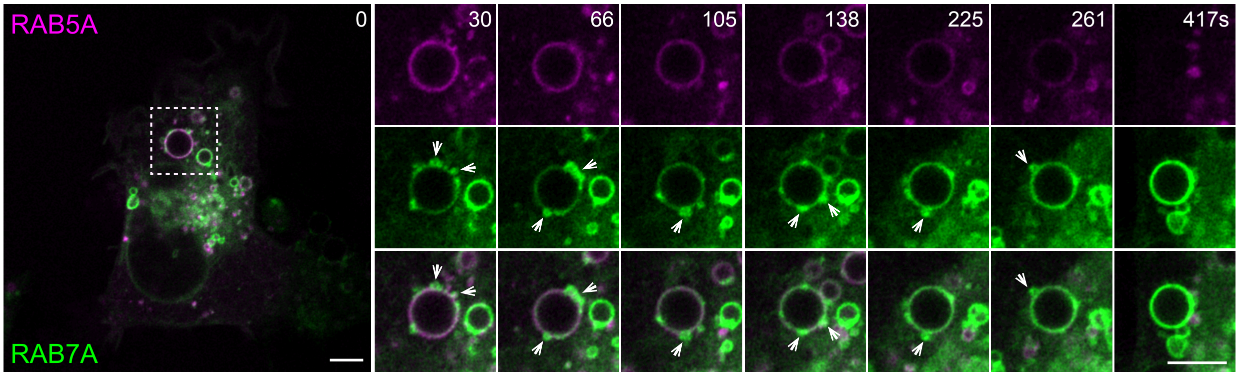

Figure S12. Macropinocytosis in HT1080 cells. Time-lapse images of the RAB5-to-RAB7 conversion on macropinosomes in HT1080 cells stably expressing mCherry-RAB5A and mEmerald-RAB7A. The arrows mark RAB7A-labeled vesicles that surrounded and fused with the RAB5A-positive macropinosome. Scale bar = 5 μm.

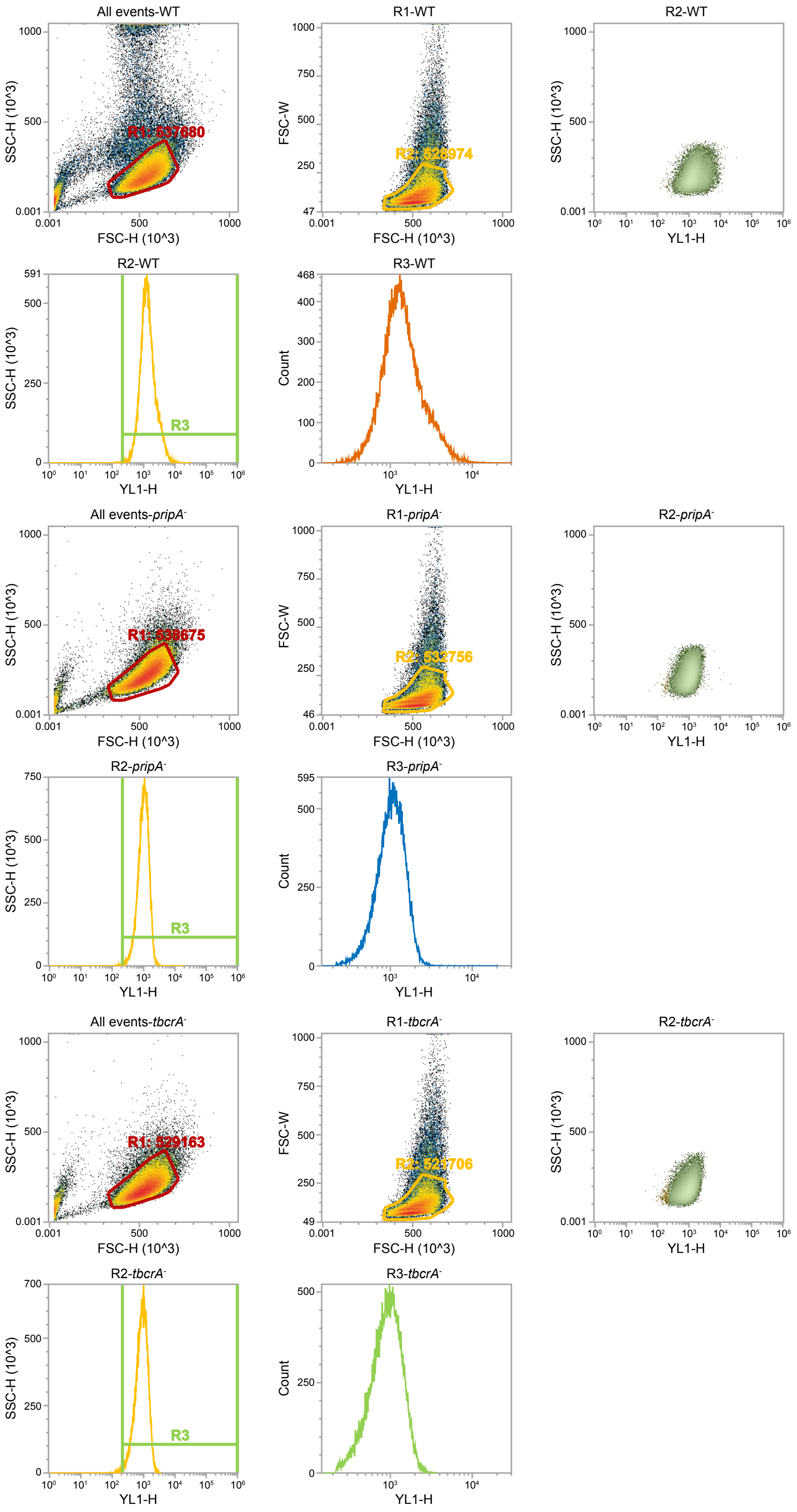

Figure S13. Gating strategies for flow cytometry analysis. Viable cells were gated by FSC-H/SSC-H scatter plots; from this gate, single cells were gated by FSC-H/FSC-W scatter plots. Cells in the single cell gate were measured for DQ-BSA fluorescence.

Table S1: Plasmids used in this study.		
**Plasmid for expression in *Dictyostelium* cells**	**References**	**Drug for selection**
pDM323-PripA	This Study	G418 (20 µg/ml)
pDM323-PripA-PH	This Study	G418 (20 µg/ml)
pDM323-PripA-△PH	This Study	G418 (20 µg/ml)
pDM451-PripA	This Study	Hygromysin (50 µg/ml)
pDM451-PripA-PH	This Study	Hygromysin (50 µg/ml)
pDM451-PripA-△PH	This Study	Hygromysin (50 µg/ml)
pDM317-TbcrA	This Study	G418 (20 µg/ml)
pDM317-TbcrA-RCC1	This Study	G418 (20 µg/ml)
pDM317-TbcrA-TBC	This Study	G418 (20 µg/ml)
pDM317-TbcrA^{R987A}	This Study	G418 (20 µg/ml)
pDM449-TbcrA-PripA	This Study	Hygromysin (50 µg/ml)
pDM449-TbcrA^{R987A}-PripA	This Study	Hygromysin (50 µg/ml)
pDM449-DDB_G0269982-PripA	This Study	Hygromysin (50 µg/ml)
pDM449-DDB_G0269982^{R322A}-PripA	This Study	Hygromysin (50 µg/ml)
pDM449-Rab5A	This Study	Hygromysin (50 µg/ml)
pDM449-Rab7A	This Study	Hygromysin (50 µg/ml)
pDM317-Rab5A	This Study	G418 (20 µg/ml)
pDM317-Rab7A	This Study	G418 (20 µg/ml)
pDM317-Rab5A^{Q68L}	This Study	G418 (20 µg/ml)
pDM317--Rab5A^{S23N}	This Study	G418 (20 µg/ml)
pDM317--Rab7A^{Q67L}	This Study	G418 (20 µg/ml)
pDM317--Rab7A^{T22N}	This Study	G418 (20 µg/ml)
pDM317-Rab5B	This Study	G418 (20 µg/ml)
pDM317-Rab7B	This Study	G418 (20 µg/ml)
pDM317-Rab32A	This Study	G418 (20 µg/ml)
pDM317-Rab32D	This Study	G418 (20 µg/ml)
pDM317-RabJ	This Study	G418 (20 µg/ml)
pDM451-VacA	This Study	Hygromysin (50 µg/ml)
pDM451-TAPP1	This Study	Hygromysin (50 µg/ml)
pDM317-2xFyve	This Study	G418 (20 µg/ml)
GRP1-PH-pDM323	Ref 38	G418 (20 µg/ml)
pDM451-EEA1$^{1-209}$	This Study	Hygromysin (50 µg/ml)
**Plasmid for expression in HT 1080 cells**		
pCDH-CMV-mCherry-RAB5A	This Study	Puromycin(10 µg/m)
pCDH-CMV-mEmerald-RAB7A	from Dr. Dong Li's Lab	Puromycin(10 µg/ml)
**Plasmid for gene knockout**		
pBluescrip-BSR		
pBluescrip-PripA(Arm1)-BSR-PripA(Arm2)	This Study	Blasticidin (10 µg/ml)
pBluescrip-TbcrA(Arm1)-BSR-TbcrA(Arm2)	This Study	Blasticidin (10 µg/ml)
pBluescrip-PIKfyve(Arm1)-BSR-PIKfyve(Arm2)	This Study	Blasticidin (10 µg/ml)
pDEX-NLS-Cre		G418 (20 µg/ml)

Plasmid for yeast two hybrid assay	
pGADT7	from Dr. Xiaochen Wang's Lab
pGBKT7	from Dr. Xiaochen Wang's Lab
pGADT7-PripA	This Study
pGADT7-PripA-△PH	This Study
pGADT7-TbcrA-TBC	This Study
pGBKT7-Rab5A(△CCN)	This Study
pGBKT7-Rab5A^{Q68L}(△CCN)	This Study
pGBKT7-Rab5A^{S23N}(△CCN)	This Study
pGBKT7-Rab7A(△CC)	This Study
pGBKT7-Rab7A^{Q67L}(△CC)	This Study
pGBKT7-Rab7A^{T22N}(△CC)	This Study
pGBKT7-Rab4(△CSC)	This Study
pGBKT7-Rab4^{Q66L}(△CSC)	This Study
pGBKT7-Rab5B	This Study
pGBKT7-Rab5B^{Q107L}	This Study
pGBKT7-Rab6(△CSSSRC)	This Study
pGBKT7-Rab6^{Q65L}(△CSSSRC)	This Study
pGBKT7-Rab7B(△CC)	This Study
pGBKT7-Rab7B^{Q61L}(△CC)	This Study
pGBKT7-Rab8A(△CC)	This Study
pGBKT7-Rab8A^{Q74L}(△CC)	This Study
pGBKT7-Rab8B(△CC)	This Study
pGBKT7-Rab8B^{Q74L}(△CC)	This Study
pGBKT7-Rab11A(△CC)	This Study
pGBKT7-Rab11A^{Q72L}(△CC)	This Study
pGBKT7-Rab11B(△CC)	This Study
pGBKT7-Rab11B^{Q70L}(△CC)	This Study
pGBKT7-Rab11C(△CC)	This Study
pGBKT7-Rab11C^{Q69L}(△CC)	This Study
pGBKT7-Rab14(△CSC)	This Study
pGBKT7-Rab14^{Q67L}(△CSC)	This Study
pGBKT7-Rab18(△CSC)	This Study
pGBKT7-Rab18^{Q65L}(△CSC)	This Study
pGBKT7-Rab21(△CCSN)	This Study
pGBKT7-Rab21^{Q66L}(△CCSN)	This Study
pGBKT7-Rab24(△CC)	This Study
pGBKT7-Rab32A(△CCK)	This Study
pGBKT7-Rab32A^{Q75L}(△CCK)	This Study
pGBKT7-Rab32B(△CCK)	This Study
pGBKT7-Rab32C(△CC)	This Study
pGBKT7-Rab32C^{Q87L}(△CC)	This Study
pGBKT7-Rab32D(△CFNCK)	This Study
pGBKT7-Rab32D^{Q68L}(△CFNCK)	This Study
pGBKT7-RabA(△CIIN)	This Study
pGBKT7-RabA^{Q67L}(△CIIN)	This Study

pGBKT7-RabC(△CC)	This Study
pGBKT7-RabC^{Q67L}(△CC)	This Study
pGBKT7-RabF(△CIIN)	This Study
pGBKT7-RabJ(△CCG)	This Study
pGBKT7-RabJ^{Q68L}(△CCG)	This Study
pGBKT7-RabL(△CC)	This Study
pGBKT7-RabL^{Q66L}(△CC)	This Study
pGBKT7-RabO(△CFIL)	This Study
pGBKT7-RabO^{Q67L}(△CFIL)	This Study
pGBKT7-RabQ(△CCK)	This Study
pGBKT7-RabQ^{Q64L}(△CCL)	This Study
**Plasmid for protein purification**	
pGEX 4T-1	
pGEX 4T-1-Rab5A^{Q68L}	This Study
pGEX 4T-1-Rab5A^{S23N}	This Study
pGEX 4T-1-Rab7A^{Q67L}	This Study
pGEX 4T-1-Rab7A^{T22N}	This Study
pGEX 6P-1-PH$^{124-219}$	This Study
pGEX 6P-1-TBC$^{820-1194}$	This Study
pGEX 6P-1-TBC$^{820-1194;mut}$	This Study
pET-MBP-3C-Rab5A(△CCN)	This Study
pET-MBP-3C-Rab7A(△CC)	This Study
pGEX 4T-1-EEA1$^{1-209}$	from Dr. Hong Zhang's Lab

Table S2: Primers used in this study. All primer sequences are given in 5' to 3' direction and each primer is designated as forward (F) or reverse (R).

Expression in *Dictyostelium* cells

Usage	Plasmid backbone	Sequence
PripA-GFP and PripA-RFP	pDM323 and pDM451	F: CGGGAGCTCAAATAAAAATGTCGTCTGTAAGAGCCCAATTTG
		R: CGGACTAGTAGCTTGATTAAATTGATTTGAAATC
PripA-PH-GFP and PripA-PH-RFP	pDM323 and pDM451	R: CGGACTAGTCTTTTGAGAATTTTGTAATGATTG
PripA-△PH-GFP and PripA-△PH-RFP	pDM323 and pDM451	F: CGGGAGCTCAAATAAAAATGCAATCATTACAAAATTCTCAAAAG
GFP-TbcrA	pDM317	F: CGGGAGCTCATGGATGACGAAGATTATAGAAATTC
		R: CGGACTAGTAATAAAATCTTTATTAATTAATAGTATAATTTTTTTAATAAATTTTGGAATATGAACGGCGGAAATTGAATC
GFP-TbcrA-RCC1	pDM317	R: CGGACTAGTACCATCAAATACATCTAAACTAC
GFP-TbcrA-TBC	pDM317	F: CGGGAGCTCATGGGTAGTTTAGATGTATTTGATG
GFP-TbcrA^{R987A}	pDM317	mutation F: GTTGATTTACCAgcAACTTTCCC
		mutation R: GGGAAAGTTgcTGGTAAATCAAC
		F: CGGGAGCTCATGGATGACGAAGATTATAGAAATTC
		R:CGGACTAGTACTAGATCCGCTAGCTTAAATA
PripA + RFP-TbcrA and PripA + RFP-TbcrA^{R987A}	pDM344 & pDM449	PripA F: CGCGGATCCATGTCGTCTGTAAGAGCCCAATTTG
		PripA R: CGGACTAGTTTAAGCTTGATTAAATTGATTTGAAATC
		TBCR1 F: CGGGAGCTCATGGATGACGAAGATTATAGAAATTC
		TBCR1 R:CGGACTAGTACTAGATCCGCTAGCTTAAATA
PripA + RFP-DDB_G0269982 and PripA + RFP-DDB_G0269982^{R322A}	pDM344 & pDM449	mutation F: CAAGATATTTTAGAAgcAATTTTATATTTATG
		mutation R: CCCCATAAATATAAAATTgcTTCTAAAATATC
		F: CGGGAGCTCATGAGTAACCATAGTGATACAAATAG
		R: CTAGCTAGCCTTTTTAATAATATTATTATTTAAATTTATATTATTACTTTTTAAATGTGATTGAGC TTCTTCATATAATG
GFP- and RFP-Rab5A	pDM317 and pDM449	F: CGGGAGCTCATGAATAATAATAATAAGATATTTC
		R: CGGACTAGTGTTACAACATTTGTTTTTCTTTCC
GFP- and RFP-Rab7A	pDM317 and pDM449	F: CGGGAGCTCATGGCCACAAAGAAAAAGG
		R: CGGACTAGTACAACAACCTGATTTAGCTGG
GFP-Rab5B	pDM317	F: CGGGAGCTCATGTCAAATGCAAATAACAATGGAC
		R: CTAGCTAGCTTTATAATTTTCAATTAATCTTCTAC
GFP-Rab7B	pDM317	F: CGGGAGCTCATGACAAAAGGAAGGAAAATTATTAAAG
		R: CTAGCTAGCACAACATGAACTTTGATTTGACTTTTTTTCTG
GFP-Rab32A	pDM317	F: CGGGAGCTCATGTCAAACAACCCAGCTGATGATG
		R: CTAGCTAGCTTTACAACAACTTGGACCAGTTGAAG
GFP-Rab32D	pDM317	F: CGGGAGCTCATGATAAATATGACAGAGACAAAAC
		R: CTAGCTAGCTTTACAATTGAAACAACTTGAAGG

GFP-RabJ	pDM317	F: CGGGAGCTCATGGATCCATTGTCAATATCAATG
		R: CTAGCTAGCACCACAACATTGATCACTATTATCAC
VacA-RFP	pDM451	F: CGGGAGCTCaaataaaaATGATTGAAGGTAGTGGTAG
		R: CGGACTAGTATTCTTTTTTTGTTGAATAG
TAPP1-GFP and TAPP1-RFP	pDM323 and pDM451	F: CGGGAGCTCATGCCTTATGTGGATCGTCAGAATC
		R: CTAGCTAGCCACGTCACTGACCGGAAGGCTCGC
**Expression in HT1080 cells**		
pCDH-CMV-mCherry-RAB5A	pCDH-CMV	RAB5A-F: TCCGGACTCAGATCTCGAGCCATGGCTAGTC GAGGCGCA
		RAB5A-R: TCCTTCGCGGCCGCGGATCCTTAGTTACTAC AACACTGATTCCTGGTTGG
		mCherry-F: GCTAGCGAATTCATGGTGAGCAATGGCGAG
		mCherry-R: AGATCTGAGTCCGGACTTGTACAGCTCGTCC ATGCC
**Generation of knockout cells**		
pripA knockout	pBluescript-BSR	Insert 1 F: cggGGTACCGTTCCCACCCCCTGGTCAAATTG
		Insert 1 R: cccAAGCTTCTAGTGGTGCTCTCTAGAATTTG
		Insert 2 F: cggACTAGTGAAATGAAGAGATGGATGCAAAG
		Insert 2 R: ataagaatGCGGCCGCGCTTGATTAAATTGATTT GAAATCC
tbcrA knockout	pBluescript-BSR	Insert 1 F: CGGGGTACCATGGATGACGAAGATTATAGAAAT TC
		Insert 1 R: CCGGAATTCGACTTGATGATGTTATTGTTGAAC
		Insert 2 F: CGGACTAGTGAGTATTCAACATTTATTAAAAC
		Insert 2 R: ATAAGAATGCGGCCGCGGAATATGAACGGCGG AAATTG
pikfyve knockout	pBluescript-BSR	Insert 1 F: CACGGTACCCAAAACTAAATATCTTTTTGATACG TG
		Insert 1 R: GAGGTCGACCTGCCATTATTAGGATTATTTGAAC
		Insert 2 F: CGGACTAGTCAAGTCCAACAAATTAATAAATAAC
		Insert 2 R: CGGCGGCCGCCATTTGATATGTTTAAATCAGAT AATGG
**Expression in AH109 (Yeast two-hybrid assay)**		
AD-PripA	pGADT7	F: CGCCATATGATGTCGTCTGTAAGAGCCCAATTTG
		R: CGCGGATCCAGCTTGATTAAATTGATTTGAAATC
AD-PripA$^{219-808}$	pGADT7	F: CGCCATATGATGCAATCATTACAAAATTCTCAAAAG
AD-TbcrA$^{743-1194}$	pGADT7	F: CGCCATATGGATAAAAGAATGAAGAACCATCACCT
		R: CGCGGATCCAATAAAATCTTTATTAATTAATAGTATAATT TTTTTAATAAATTTTGGAATATGAAC GGCGGAAATTGAATC
BD-Rab5A(△CCN)	pGBKT7	F:CGCCATATGATGAATAATAATAATAAGATATTTC
		R:CGCGGATCCTTTGTTTTTCTTTCCAGTGTTACC
BD-Rab5A^{Q68L}(△CCN)	pGBKT7	mutation F: CAGCTGGTttAGAACGTTATCAT
		mutation R: ATAACGTTCAaaACCAGCTGTATC

BD-Rab5A^{S23N}(△CCN)	pGBKT7	mutation F: GTAGGCAAAAaTTCTTTAGTATTGA
		mutation R: TCAATACTAAAGAAtTTTTGCCTAC
BD-Rab7A(△CC)	pGBKT7	F: CGCCATATGATGGCCACAAAGAAAAAGG
		R: CGCGGATCCACCTGATTTAGCTGGTTGTGGTTC
BD-Rab7A^{Q67L}(△CC)	pGBKT7	mutation F: GATACAGCTGGTttAGAACGTTTC
		mutation R: TAATGATTGGAAACGTTCTaaACCAGC
BD-Rab7A^{T22N}(△CC)	pGBKT7	mutation F: GGTGTTGGTAAAAatTCTCTCATGAATCAAT
		mutation R: GAGAGAatTTTTACCAACACCTGAATC
BD-Rab4(△CSC)	pGBKT7	F: CGCCATATGATGGGAAAACCTTTATATCTTG
		R: CGCGGATCCATTAGATGAATCACCAGGTTTCTTTG
BD-Rab4^{Q66L}(△CSC)	pGBKT7	mutation F: GATACTGCCGGACtAGAAAGATTTAG
		mutation R: CTAAATCTTTCTaGTCCGGCAGTATC
BD-Rab5B	pGBKT7	F: CGCCATATGATGTCAAATGCAAATAACAATGGAC
		R: CGCGGATCCTTTATAATTTTCAATTAATCTTCTAC
BD-Rab5B^{Q107L}	pGBKT7	mutation F: GATACTGCAGGACtAGAACGTTATAG
		mutation R: CTATAACGTTCTaGTCCTGCAGTATC
BD-Rab6(△CSSSRC)	pGBKT7	F: CGCCATATGATGGAATCATTATCAAAATATAAAT
		R: CGCGGATCCAAATCCATCACCTTCTTTTAAGGC
BD-Rab6^{Q65L}(△CSSSRC)	pGBKT7	mutation F: GATACTGCAGGTCtAGAAAGATTTAG
		mutation R: CTAAATCTTTCTaGACCTGCAGTATC
BD-Rab7B(△CC)	pGBKT7	F: CGCCATATGATGACAAAAGGAAGGAAAATTATTAAAG
		R: CGCGGATCCTGAACTTTGATTTGACTTTTTTTCTG
BD-Rab7B^{Q61L}(△CC)	pGBKT7	mutation F: CACAAGTGGTCtAGAGAGATTCAG
		mutation R: CTGAATCTCTCTaGACCACTTGTG
BD-Rab8A(△CC)	pGBKT7	F: CGCCATATGATGACTTCTCCAGCAACAAATAAATC
		R: CGCGGATCCAGCTTTTTTCTTATTGTTATTTGCACC
BD-Rab8A^{Q74L}(△CC)	pGBKT7	mutation F: GGATACTGCAGGTCtAGAAAGATTCAG
		mutation R: CTGAATCTTTCTaGACCTGCAGTATCC
BD-Rab8B(△CC)	pGBKT7	F: CCGGAATTCATGACTTCTCCAGCAACAAATAAAC
		R: CGCGGATCCAGTATTTTTCTTATTGTTTGGAGTAATG
BD-Rab8B^{Q74L}(△CC)	pGBKT7	mutation F: GACACTGCAGGTCtAGAAAGATTCAG
		mutation R: CTGAATCTTTCTaGACCTGCAGTGTC
BD-Rab11A(△CC)	pGBKT7	F: CGCCATATGATGACTTCAAAAGGATCACAAG
		R: CGCGGATCCACCAGATTTGGCGGCTGGTGGTTC
BD-Rab11A^{Q72L}(△CC)	pGBKT7	mutation F: GGATACTGCAGGTCtAGAAAGATATAG
		mutation R: CTATATCTTTCTaGACCTGCAGTATCC
BD-Rab11B(△CC)	pGBKT7	F: CGCCATATGATGGTACTAAAAACAATAGAATATG
		R: CGCGGATCCATCATATACTGGTTTATTAATTG
BD-Rab11B^{Q70L}(△CC)	pGBKT7	mutation F: GATACTGCAGGTCtAGAAAAATATAATTC
		mutation R: GAATTATATTTTTCTaGACCTGCAGTATC
BD-Rab11C(△CC)	pGBKT7	F: CGCCATATGATGCCACAAGAAGAAGAAGCAG
		R: CGCGGATCCACCACTTTTCTTTTCTTGATGAG
BD-Rab11C^{Q69L}(△CC)	pGBKT7	mutation F: GATACTGCAGGTCtAGAAAGATTTAG
		mutation R: CTAAATCTTTCTaGACCTGCAGTATC
BD-Rab14(△CSC)	pGBKT7	F: CCGGAATTCATGTCATTTCCATATGAATATATAT
		R: CGCGGATCCTTTACTGGCATCTTGAGGTTTATCAG

BD-Rab14^{Q67L}(△CSC)	pGBKT7	mutation F: GATACTGCAGGTCtAGAAAGATTCAGG<br>mutation R: CCTGAATCTTTCTaGACCTGCAGTATC
BD-Rab18(△CSC)	pGBKT7	F: CCGGAATTCATGGAAGAAGATAAACAATATAAAG<br>R: CGCGGATCCAACACCTTGATTATGATCAGGTTC
BD-Rab18^{Q65L}(△CSC)	pGBKT7	mutation F: GGATACTGCAGGACtAGAGAAATTTAG<br>mutation R: CTAAATTTCTCTaGTCCTGCAGTATCC
BD-Rab21(△CCSN)	pGBKT7	F: CGCCATATGATGACAGATACTGAAAAAAGTTTTAAAG<br>R: CGCGGATCCACCTGGTTGTTTATTACCTGAATC
BD-Rab21^{Q66L}(△CCSN)	pGBKT7	mutation F: GATACAGCAGGACtAGAAAGATTTCATG<br>mutation R: CATGAAATCTTTCTaGTCCTGCTGTATC
BD-Rab24(△CC)	pGBKT7	F: CGCCATATGATGACAAAGACAAAAATTGATC<br>R: CGCGGATCCGCCACCTTTTTTCTTTTGAGTTTG
BD-Rab32A(△CCK)	pGBKT7	F: CGCCATATGATGTCAAACAACCCAGCTGATGATG<br>R: CCGGAATTCACTTGGACCAGTTGAAGTTGGTTG
BD-Rab32A^{Q75L}(△CCK)	pGBKT7	mutation F: GATATTGCAGGTCtAGAAAGATTTGG<br>mutation R: CCAAATCTTTCTaGACCTGCAATATC
BD-Rab32B(△CCK)	pGBKT7	F: CGCCATATGATGAATAGAGGTGATATATTTGC<br>R: CGCGGATCCTGATTTTGAATCATCAGATTTCTC
BD-Rab32C(△CC)	pGBKT7	F: CGCGAATTCATGTATAGTAATAAAAATGATAAAG<br>R: CGCGGATCCAGTTTTTGATTGAGTTGGTGTTG
BD-Rab32C^{Q87L}(△CC)	pGBKT7	mutation F: GATATAGCAGGCCtAGAAAGATTTGG<br>mutation R: CCAAATCTTTCTaGGCCTGCTATATC
BD-Rab32D(△CFNCK)	pGBKT7	F: CGCCATATGATGATAAATATGACAGAGACAAAAC<br>R: CGCGGATCCACTTGAAGGTGAGGAAGTTGAAGG
BD-Rab32D^{Q68L}(△CFNCK)	pGBKT7	mutation F: GATACAGCAGGACtAGAGAAATATTGG<br>mutation R: CCAATATTTCTCTaGTCCTGCTGTATC
BD-RabA(△CIIN)	pGBKT7	F: CGCCATATGATGAGTAAAAAATATGAACATT<br>R: CGCGGATCCATTACTTTTTTGGGATTGAGGTTT
BD-RabA^{Q67L}(△CIIN)	pGBKT7	mutation F: GATACTGCTGGACtAGAACGATTTAG<br>mutation R: CTAAATCGTTCTaGTCCAGCAGTATC
BD-RabC(△CC)	pGBKT7	F: CGCCATATGATGGAAGAAGAAATTTTATATAAAA<br>R: CGCGGATCCGCCTCTCTTTTGACCAACTTCTG
BD-RabC^{Q67L}(△CC)	pGBKT7	mutation F: CACTGCAGGTCtAGAAAGATTCAAATC<br>mutation R: GATTTGAATCTTTCTaGACCTGCAGTG
BD-RabF(△CIIN)	pGBKT7	F: CGCCATATGATGAGTAAAGAATATGAACACTTATTC<br>R: CGCGGATCCATTATTTTTTTCGGGGGATTGTGTTTTTTT TAGAG
BD-RabJ(△CCG)	pGBKT7	F: CGCCATATGATGGATCCATTGTCAATATCAATG<br>R: CCGGAATTCTTGATCACTATTATCACTATAATTTG
BD-RabJ^{Q68L}(△CCG)	pGBKT7	mutation F: GATAGTGCTGGACtAGATAGATTTCG<br>mutation R: CGAAATCTATCTaGTCCAGCACTATC
BD-RabL(△CC)	pGBKT7	F: CGCCATATGATGAAAGAAGAAAAAACAATGTTAAAG<br>R: CGCGGATCCTGTTCCAACTTTGACTTCTTCCGG
BD-RabL^{Q66L}(△CC)	pGBKT7	mutation F: GATACCTGTGGTCtAGAACGTTTTCAAG<br>mutation R: CTTGAAAACGTTCTaGACCACAGGTATC

BD-RabO(△CFIL)	pGBKT7	F: CGCCATATGATGGATATAGATAATTCTATTTTA
		R: CGCGGATCCCATTCCTTTATTTTTTTCATTTTG
BD-RabO^{Q67L}(△CFIL)	pGBKT7	mutation F: GATACTGGAGGTCtAGAAAGATTTAAAAC
		mutation R: GTTTTAAATCTTTCTaGACCTCCAGTATC
BD-RabQ(△CCK)	pGBKT7	F: CGCCATATGATGGAAGAATATCATTTAAAGTG
		R: CGCGGATCCTTCACTCTTTGGTTTCTTTGGGGT
BD-RabQ^{Q64L}(△CCL)	pGBKT7	mutation F: GATACAGCAGGTCtAGAATCATTTAG
		mutation R: CTAAATGATTCTaGACCTGCTGTATC
**Expression in *E. coli***		
GST-Rab5A^{Q68L} and -Rab5A^{S23N}	pGEX-4T-1	F: CGCGGATCCATGAATAATAATAATAAGATATTTC
		R: ATAAGAATGCGGCCGCGTTACAACATTTGTTTTTCTT TCC
GST-Rab7A^{Q67L} and -Rab7A^{T22N}	pGEX-4T-1	F: CGCGGATCCATGGCCACAAAGAAAAAGG
		R: ATAAGAATGCGGCCGCACAACAACCTGATTTAGCTGG
GST-$PH^{124-219}$	pGEX-6P-1	F: CCGGAATTCATGCCACCGATTCTCAGTGGTTACTTAAAG
		R: CCGCTCGAGTTATTGATAATACTTTTTAAATTCTTTT AAAC
GST-TBC and GST-TBC^{mut}	pGEX-6P-1	F: CCGGAATTCATGGGCGGCTTTGAAACCATTAAG
		R: CCGCTCGAGTTAAATAAAATCCTTATTG
GST-TBC^{mut}	pGEX-6P-1	mutation F1: GATCTGCCGgcgACCTTTCCGCAG
		mutation R1: GGAAAGGTcgcCGGCAGATCCACC
		mutation F2: GATTGGTTATGTTgcGGGTATGAG
		mutation R2: GATAGCTCATACCCgcAACATAAC
His-MBP-Rab5A	pET-MBP-3C	F: CGCGGATCCATGAATAATAATAATAAGATATTTC
		R: ATAAGAATGCGGCCGCTTAGTTTTTGTTTTTCTTTC CAGTGTTACC
His-MBP-Rab7A	pET-MBP-3C	F: CGCGGATCCATGGCCACAAAGAAAAAGG
		R: ATAAGAATGCGGCCGCTTAACCTGATTTAGCTGGTT GTGGTTC

**Table S3: GAP proteins tested by Y2H.**		
**Gene number/name**	**Truncation used in Y2H**	**Gene information**
DDB_G0275421	754-945 aa	contains 2 GRAM domains, one RabGAP/TBC domain, and one EF hand domain
DDB_G0295717	85-332 aa	RabGAP/TBC domain-containing protein, calcium-binding EF-hand domain-containing protein, similar to TBC1 domain family member GTPase-activating proteins
DDB_G0269982	237-462 aa	TBC domain protein, putative Rab GTPase-activating protein, similar to human TBC1D22B and *S. pombe* gyp1
DDB_G0288811	530-748 aa	very similar to TBC1 domain family member 15 proteins and yeast GYP7
DDB_G0283055	40-223 aa	very similar to the mammalian TBC1 domain family member 20 protein, a potential multi-pass membrane protein
tbck	657-844 aa	similar to TBCK in human, fly, and worm; unlikely to function as a kinase as it does not contain a catalytic aspartate
DDB_G0288949	47-401 aa	TBC1 domain family member 7
DDB_G0279511	659-1011 aa	RabGAP/TBC domain-containing protein
DDB_G0281137	77-303 aa	RabGAP/TBC domain-containing protein
DDB_G0278601	289-390 aa	RabGAP/TBC domain-containing protein
DDB_G0270856	505-723 aa	RabGAP/TBC domain-containing protein, GRAM domain-containing protein
bub2	63-244 aa	RabGAP/TBC domain-containing protein, putative mitotic checkpoint protein
DDB_G0289735	154-346 aa	RabGAP/TBC domain-containing protein
DDB_G0287379	790-979 aa	ankyrin repeat-containing protein, RabGAP/TBC domain-containing protein
DDB_G0277967	133-321 aa	RabGAP/TBC domain-containing protein
DDB_G0288405	290-480 aa	RabGAP/TBC domain-containing protein, TLDc domain-containing protein
DDB_G0280253	374-729 aa	RabGAP/TBC domain-containing protein
DDB_G0283285	1050-1386 aa	RabGAP/TBC domain-containing protein
DDB_G0278181	641-830 aa	ankyrin repeat-containing protein, RabGAP/TBC domain-containing protein
DDB_G0282303	523-712 aa	ankyrin repeat-containing protein, RabGAP/TBC domain-containing protein
DDB_G0284995	30-229 aa	ankyrin repeat-containing protein, RabGAP/TBC domain-containing protein
DDB_G0290597	29-198 aa	RabGAP/TBC domain-containing protein, B-box zinc finger-containing protein
DDB_G0276961	271-457 aa	C-terminus similar to *A. thaliana* plant adhesion molecule 1, *D. melanogaster* extracellular matrix adhesion protein Pollux, and human rab6 GAP GAPCENA
