# Supplementary material for: The PripA-TbcrA complex-centered Rab GAP cascade facilitates macropinosome maturation in Dictyostelium
Source: Nat Commun. 2022 Apr 4;13:1787. doi: 10.1038/s41467-022-29503-1 (PMC8980073; doi:10.1038/s41467-022-29503-1)
Supplement: Supplementary file 3 — Description of Additional Supplementary Files [file 41467_2022_29503_MOESM3_ESM.pdf]

**Description of Additional Supplementary Files**

File Name: Supplementary Movie 1
Description: The Rab5- to-Rab7 conversion during macropinocytosis in Dictyostelium. Corresponds to Figure 1e. Images were captured at 3 sec per frame and played back at 5 frames per second. Scale bar = 5 µm.

File Name: Supplementary Movie 2
Description: Time-lapse imaging of GFP-Rab5A$^{REMI}$/WT cells pre-incubated with DQ-BSA. Images were acquired after DQ-BSA was washed out. Corresponds to Figure 1f. Images were captured at 3 sec per frame and played back at 5 frames per second. Scale bar = 5 µm.

File Name: Supplementary Movie 3
Description: Localization of GFP-tagged PripA-PH, PripA-ΔPH, and PripA during macropinocytosis. Corresponds to Figures 2b, c, and d. Images were captured at 6 sec per frame and played back at 3 frame per second. Scale bar = 5 µm.

File Name: Supplementary Movie 4
Description: Localization of GFP-Rab5A$^{REMI}$ and PripA-RFP during macropinocytosis. Corresponds to Figure 5d. Images were captured at 3 sec per frame and played back at 5 frames per second. Scale bar = 5 µm.

File Name: Supplementary Movie 5
Description: Localization of GFP-Rab7A and PripA-RFP during macropinocytosis. Corresponds to Figure 5e. Images were captured at 3 sec per frame and played back at 5 frames per second. Scale bar = 5 µm.

File Name: Supplementary Movie 6
Description: Colocalization of PripA-RFP and GFPTbcrA during macropinocytosis. Corresponds to Figure 6d. Images were captured at 6 sec per frame and played back at 3 frames per second. Scale bar = 5 µm.

File Name: Supplementary Movie 7
Description: The Rab5- to-Rab7 conversion during phagocytosis in Dictyostelium. Corresponds to Supplementary Figure S9a. Images were captured at 6 sec per frame and played back at 3 frames per second. Scale bar = 5 µm.

File Name: Supplementary Movie 8
Description: Localization of PripA-GFP during phagocytosis. Corresponds to Supplementary Figure S9b. Images were captured at 6 sec per frame and played back at 5 frame per second. Scale bar = 5 µm.

File Name: Supplementary Movie 9
Description: . The Rab5- to-Rab7 conversion during macropinocytosis in HT1080 cells. Corresponds to Supplementary Figure S12. Images were captured at 6 sec per frame and played back at 20 frames per second. Scale bar = 5 µm.
