# Supplementary material for: The PripA-TbcrA complex-centered Rab GAP cascade facilitates macropinosome maturation in Dictyostelium
Source: Nat Commun. 2022 Apr 4;13:1787. doi: 10.1038/s41467-022-29503-1 (PMC8980073; doi:10.1038/s41467-022-29503-1)
Supplement: Supplementary file 13 — Reporting Summary [file 41467_2022_29503_MOESM13_ESM.pdf]

nature portfolio

Corresponding author(s): Huaqing Cai

Last updated by author(s): Feb 21, 2022

# Reporting Summary

Nature Portfolio wishes to improve the reproducibility of the work that we publish. This form provides structure for consistency and transparency in reporting. For further information on Nature Portfolio policies, see our Editorial Policies and the Editorial Policy Checklist.

## Statistics

For all statistical analyses, confirm that the following items are present in the figure legend, table legend, main text, or Methods section.

| n/a | Confirmed | |
|---|---|---|
| ☐ | ☒ | The exact sample size (*n*) for each experimental group/condition, given as a discrete number and unit of measurement |
| ☐ | ☒ | A statement on whether measurements were taken from distinct samples or whether the same sample was measured repeatedly |
| ☐ | ☒ | The statistical test(s) used AND whether they are one- or two-sided<br>*Only common tests should be described solely by name; describe more complex techniques in the Methods section.* |
| ☒ | ☐ | A description of all covariates tested |
| ☒ | ☐ | A description of any assumptions or corrections, such as tests of normality and adjustment for multiple comparisons |
| ☐ | ☒ | A full description of the statistical parameters including central tendency (e.g. means) or other basic estimates (e.g. regression coefficient) AND variation (e.g. standard deviation) or associated estimates of uncertainty (e.g. confidence intervals) |
| ☐ | ☒ | For null hypothesis testing, the test statistic (e.g. *F*, *t*, *r*) with confidence intervals, effect sizes, degrees of freedom and *P* value noted<br>*Give P values as exact values whenever suitable.* |
| ☒ | ☐ | For Bayesian analysis, information on the choice of priors and Markov chain Monte Carlo settings |
| ☒ | ☐ | For hierarchical and complex designs, identification of the appropriate level for tests and full reporting of outcomes |
| ☐ | ☒ | Estimates of effect sizes (e.g. Cohen's *d*, Pearson's *r*), indicating how they were calculated |

*Our web collection on statistics for biologists contains articles on many of the points above.*

## Software and code

Policy information about availability of computer code

| | |
|---|---|
| Data collection | Image acquisition was performed using Zen black 2.1 (Zeiss).<br>Flow cytometry data was collected using Attune NxT 3.2.1 (Invitrogen). |
| Data analysis | Data analysis, statistics, and plotting were performed using Graphpad Prism 9.0.<br>Imaging data was analyzed using ImageJ 1.45j.<br>Flow cytometry analysis was performed using Attune NxT 3.2.1 (Invitrogen). |

For manuscripts utilizing custom algorithms or software that are central to the research but not yet described in published literature, software must be made available to editors and reviewers. We strongly encourage code deposition in a community repository (e.g. GitHub). See the Nature Portfolio guidelines for submitting code & software for further information.

## Data

Policy information about availability of data

All manuscripts must include a data availability statement. This statement should provide the following information, where applicable:

- Accession codes, unique identifiers, or web links for publicly available datasets
- A description of any restrictions on data availability
- For clinical datasets or third party data, please ensure that the statement adheres to our policy

The mass spectrometry data are available via ProteomeXchange with identifier PXD032072. Other supporting data are available within the paper and its supplementary information files. The underlying raw data is supplied in the "Source Data" file. Raw images and other materials are available from the corresponding author upon request.

# Field-specific reporting

Please select the one below that is the best fit for your research. If you are not sure, read the appropriate sections before making your selection.

☒ Life sciences ☐ Behavioural & social sciences ☐ Ecological, evolutionary & environmental sciences

For a reference copy of the document with all sections, see nature.com/documents/nr-reporting-summary-flat.pdf

# Life sciences study design

All studies must disclose on these points even when the disclosure is negative.

| | |
|---|---|
| Sample size | No statistical methods were used to pre-determine the correct sample size. All statistical calculations were derived from at least 3 biological replicates. For microscopy-based assays, each biological repeat included sufficient cell numbers to ensure that the effects could be robustly measured. |
| Data exclusions | Live cell imaging data was excluded only if the imaged cells showed phototoxicity effects or migrated out of the imaging field, or the imaged subjects moved out of the focal plane. In these cases, samples were excluded prior to analysis. |
| Replication | Data was replicated in independent experiments (at least two independent experiments ). |
| Randomization | This study used cultured cell lines which are homogenous and isogenic, therefore, no randomization was used prior to treatment. |
| Blinding | Investigators were not blinded. Key experiments were repeated by independent researchers. To reduce potential human bias, quantitative measurements were performed and cells were selected before their behavior was known. |

# Reporting for specific materials, systems and methods

We require information from authors about some types of materials, experimental systems and methods used in many studies. Here, indicate whether each material, system or method listed is relevant to your study. If you are not sure if a list item applies to your research, read the appropriate section before selecting a response.

## Materials & experimental systems

| n/a | Involved in the study | |
|---|---|---|
| ☐ | ☒ | Antibodies |
| ☐ | ☒ | Eukaryotic cell lines |
| ☒ | ☐ | Palaeontology and archaeology |
| ☒ | ☐ | Animals and other organisms |
| ☒ | ☐ | Human research participants |
| ☒ | ☐ | Clinical data |
| ☒ | ☐ | Dual use research of concern |

## Methods

| n/a | Involved in the study | |
|---|---|---|
| ☒ | ☐ | ChIP-seq |
| ☐ | ☒ | Flow cytometry |
| ☒ | ☐ | MRI-based neuroimaging |

## Antibodies

| | |
|---|---|
| Antibodies used | GFP antibody (WB 1:5000, Roche #11814460001), DsRed antibody (WB 1:1000, TaKaRa #632496) |
| Validation | Antibodies were validated using Dictyostelium cells lysates expressing GFP- or RFP-fusion proteins. |

## Eukaryotic cell lines

Policy information about cell lines

| | |
|---|---|
| Cell line source(s) | Dictyostelium Ax2 cells from Robert Kay Lab (MRC Laboratory of Molecular Biology, London, UK), HEK293T cells from Li Yu Lab (Tsinghua University, Beijing, China), and HT1080 cells from ATCC. |
| Authentication | Cell lines used were not authenticated. |
| Mycoplasma contamination | Cell lines were tested negative for mycoplasma contamination. |
| Commonly misidentified lines (See ICLAC register) | No commonly misidentified cell lines were used in the study. |

## Flow Cytometry

### Plots

Confirm that:

☒ The axis labels state the marker and fluorochrome used (e.g. CD4-FITC).

☒ The axis scales are clearly visible. Include numbers along axes only for bottom left plot of group (a 'group' is an analysis of identical markers).

☒ All plots are contour plots with outliers or pseudocolor plots.

☒ A numerical value for number of cells or percentage (with statistics) is provided.

### Methodology

| | |
|---|---|
| Sample preparation | 2-3x106 cells seeded in 6-well plate were incubated in SIH medium supplemented with 10 ug/ml DQ-BSA for 20 min. After incubation, cells were washed with ice-cold KK2 buffer (6.5 mM KH2PO4, 3.8 mM K2HPO4, pH 6.2) containing 10 mM EDTA and resuspended in KK2 containing 5 mM sodium azide. |
| Instrument | Attune NxT flow cytometer (ThermoFisher) |
| Software | Attune NxT 3.2.1 (Invitrogen) |
| Cell population abundance | No sorting was performed, only gating for measurements. |
| Gating strategy | Viable cells were gated by FSC-H/SSC-H scatter plots; from this gate, single cells were gated by FSC-H/FSC-W scatter plots. Cells in the "single cell" gate were measured for DQ-BSA fluorescence. Details of the gating strategy is shown in supplementary figure S13. |

☒ Tick this box to confirm that a figure exemplifying the gating strategy is provided in the Supplementary Information.
